# The effect of Zhizi Qinggan decoction in treating hyperthyroidism: A systematic review and meta-analysis

**DOI:** 10.1097/MD.0000000000044483

**Published:** 2025-09-12

**Authors:** Min Zhang, Jiashuai Chu, Chang Xiu, Jing Bai, Lei Fan

**Affiliations:** a Discipline Inspection Commission Office, The Third Affiliated Hospital of Heilongjiang University of Chinese Medicine, Harbin, China; b Graduate School, Heilongjiang University of Chinese Medicine, Harbin, China; c Department of Gastrointestinal Surgery, The Third Affiliated Hospital of Heilongjiang University of Chinese Medicine, Harbin, China.

**Keywords:** hyperthyroidism, meta-analysis, Zhizi Qinggan decoction

## Abstract

**Background:**

This study aims to systematically assess the efficacy of Zhizi Qinggan Decoction combined with methimidazole tablets for the treatment of hyperthyroidism through a systematic review and meta-analysis, to provide evidence for clinical practice.

**Methods:**

A computer search of both Chinese and English databases, including CNKI, WangFang, VIP, CBM, PubMed, Cochrane Library, Embase, and Web of Science, was conducted. The search period was from the establishment of the database to August 2024. Randomized controlled trials (RCTs) related to the treatment of hyperthyroidism with Zhizi Qinggan Decoction were collected. Two independent researchers conducted the literature search, selection, and quality assessment. A meta-analysis was conducted using Stata 14.0 software to evaluate various outcome indicators, including thyroid hormone levels, clinical effectiveness, and traditional Chinese medicine syndrome scores.

**Results:**

A total of 16 RCTs were included, involving 1320 patients with hyperthyroidism with an average age of 39.3 years. The meta-analysis results indicated that Zhizi Qinggan Decoction combined with Western medicine significantly improved outcomes in the treatment of hyperthyroidism compared to Western medicine alone. Specifically, the results for free triiodothyronine (standardized mean difference [SMD] = –0.79; 95% confidence interval [CI]: –1.11, –0.47; *P* < .0001), free thyroxine (SMD = –2.09; 95% CI: –2.91, –1.28; *P* < .0001), thyroid-stimulating hormone (SMD = 1.99; 95% CI: 1.00, 2.99; *P* < .0001), thyroid volume (SMD = –1.22; 95% CI: –1.84, –0.60; *P* < .001), clinical efficacy (odds ratio = 6.05; 95% CI: 3.85, 9.51; *P* < .0001), adverse reactions (odds ratio = 2.61; 95% CI: 1.52, 4.45; *P* < 0.001), and traditional Chinese medicine syndrome score (SMD = –1.85; 95% CI: –2.41, –1.28; *P* < .001) all showed statistically significant improvements (*P* < .001).

**Conclusion:**

Current evidence suggests that the combination of Zhizi Qinggan Decoction and methimazole tablets provides good clinical therapeutic outcomes. However, further efforts are needed to strengthen the evidence supporting its clinical application through rigorous large-sample RCTs.

## 1. Introduction

Hyperthyroidism is a common endocrine disorder, primarily characterized by the disruption of thyroid hormone levels and changes in related signaling pathways, which can lead to severe pathological conditions.^[1,2]^ It mainly causes changes in the levels of TSH, FT3, and FT4 hormones in the body. TSH is secreted by the pituitary gland and negatively regulates the synthesis and release of thyroid hormones (T3/T4) to maintain the balance of thyroid function. FT3 directly participates in cellular metabolism, heart rate, and body temperature regulation. FT4 exerts biological activity after being converted into FT3 and is involved in the metabolism of fats, proteins, and carbohydrates. In hyperthyroidism, excessive secretion of FT3/FT4 leads to an increased heart rate and a rise in basal metabolic rate. However, excessive FT3/FT4 can inhibit the secretion of TSH by the pituitary gland, further reducing the synthesis of thyroid hormones and forming a self-regulating closed loop.^[3,4]^ Clinically, it manifests as fatigue, palpitations, tremors, irritability, exophthalmos, and thyroid enlargement, significantly affecting the patient’s physical health and quality of life.^[5]^ Studies indicate that 0.1% to 2.5% of the population generally has hyperthyroidism, with Graves’ disease being the most common, accounting for approximately 80% of hyperthyroidism cases. The pathogenesis remains unclear and is mainly associated with immune, genetic, and environmental factors.^[6–8]^ Modern medical treatments for hyperthyroidism primarily include antithyroid medications, radioactive iodine therapy, and surgical treatment.^[9]^ The use of antithyroid medications requires frequent monitoring of thyroid function, has a long treatment duration, high recurrence rates, and can lead to adverse reactions such as liver damage and leukopenia.^[10]^ Radioactive iodine can cause irreversible damage to thyroid follicular epithelial cells, and surgery carries a high risk of complications such as thyroid dysfunction.^[11]^ Therefore, seeking safe and effective alternative therapies has become an important direction in clinical research in recent years.

Traditional Chinese medicine (TCM), guided by the concept of holistic treatment, emphasizes the principles of syndrome differentiation and comprehensive treatment, gradually demonstrating unique advantages in the treatment of hyperthyroidism.^[12]^ TCM holds that the occurrence of hyperthyroidism is closely related to the liver, with the fundamental pathogenesis being qi stagnation, phlegm accumulation, and blood stasis in the anterior neck.^[13]^ The TCM treatment principle focuses on soothing the liver and relieving stagnation, clearing the liver, and draining fire. Zhizi Qinggan Decoction, derived from “Wai Ke Zheng Zong,” includes key ingredients such as gardenia, peony root bark, bupleurum, angelica, paeony, poria, chuanxiong, burdock seed, and licorice. It is used to soothe the liver, regulate qi, cool the blood, clear heat, and drain fire to reduce swelling.^[14]^ The main active ingredients include iridoid glycosides, saikosaponins, paeoniflorin, and ligustilide. These components exert therapeutic effects through multiple pathways: for example, the high-polarity iridoid glycosides in gardenia can reduce the levels of serum thyroid hormones (T3, T4) in hyperthyroid models by regulating the expression of genes related to thyroid hormone synthesis; saikosaponins may regulate the secretion of thyroid-stimulating hormone (TSH) by modulating the hypothalamic-pituitary-thyroid axis; paeoniflorin has anti-inflammatory and immunomodulatory effects, which may alleviate the autoimmune reactions involved in the pathogenesis of hyperthyroidism.^[15–18]^ Numerous studies on the use of Zhizi Qinggan Decoction in treating thyroid diseases, particularly hyperthyroidism, indicate that when combined with conventional Western medicines such as Methimazole tablets, it can improve thyroid function indicators (e.g., reducing FT3 and FT4 levels, increasing TSH levels), alleviate TCM syndrome symptoms (e.g., palpitations, irritability, sweating), enhance clinical efficacy, and reduce the incidence of adverse reactions compared to Western medicine alone. However, these studies vary in sample size, scientific rigor of study design, and consistency of results, leading to an incomplete, inconsistent, and inaccurate understanding of the exact efficacy of this combined treatment approach.^[18–33]^ Moreover, no scholars have conducted a meta-analysis on this topic before. Therefore, conducting a meta-analysis of the effect of Zhizi Qinggan Decoction combined with Methimazole tablets in treating hyperthyroidism is of great significance. This will provide more scientific and reliable evidence for the clinical treatment of hyperthyroidism patients.

## 2. Materials and methods

This study was strictly conducted according to the PRISMA guidelines^[20]^ and has been registered on the PROSPERO platform (registration number: CRD42024614122).

### 2.1. Inclusion criteria

Study type: RCTs on the treatment of hyperthyroidism with Zhizi Qinggan Decoction; study population: Patients who meet the diagnostic criteria for hyperthyroidism based on clinical symptoms and examinations, with no restrictions on age, gender, or disease duration; diagnostic criteria: Based on the authoritative diagnostic standards for hyperthyroidism in the “Guidelines for Clinical Research of New Chinese Medicines”^[21]^; experimental group: Zhizi Qinggan Decoction combined with Methimazole tablets, control group: Methimazole tablets; primary outcome measures: Serum thyroid hormone levels (FT3, FT4, TSH), clinical total efficacy rate (cure rate + marked efficacy rate + effective rate); secondary outcome measures: TCM syndrome score (TCM syndrome score in this review is a quantitative assessment tool for evaluating the severity of TCM-related symptoms in hyperthyroidism patients. It includes specific symptoms such as dizziness, palpitations, irritability, finger tremors, sweating, insomnia, and fatigue. These symptoms are scored based on their presence and severity, with the total score reflecting the overall status of the patient’s TCM syndrome.), thyroid volume, adverse reactions.

In this study, Methimazole Tablets are the only Western medicine included for treatment. Although the treatment of hyperthyroidism covers various drug categories (such as thioamides, beta-blockers, etc), Methimazole is usually the first choice due to its lower risk of hepatotoxicity, longer half-life, and the convenience of once-daily administration.^[19]^ Additionally, to ensure the consistency of interventions in the control groups of each combined trial, when including studies on the combined treatment with Zhizi Qinggan Decoction, relevant studies involving the combination of Zhizi Qinggan Decoction with non-Methimazole regimens have been excluded.

### 2.2. Exclusion criteria

Duplicate publications; non-randomized controlled trials; studies where the data could not be extracted, and attempts to contact the original authors were unsuccessful; animal studies; patients who received other drug treatments.

### 2.3. Literature search

A computer search was performed in CNKI, WangFang, VIP, CBM, PubMed, Embase, Web of Science, and Cochrane Library databases. The search terms used were a combination of subject terms and free terms, including “Zhizi Qinggan Decoction,” “Hyperthyroidism,” “Hyperthyroid,” “Graves Disease,” “Hyperthyroids,” and “Primary Hyperthyroidism.” The search period was from the establishment of the database to August 2024. As an example, the specific search strategy used for PubMed is provided in Supplementary File 1, Supplemental Digital Content, https://links.lww.com/MD/P943.

### 2.4. Literature screening and data extraction

Two researchers performed the literature search. The search results were imported into NoteExpress to eliminate duplicate publications. Then, based on the inclusion and exclusion criteria, the titles and abstracts were reviewed for initial screening, followed by a full-text screening. After determining the final studies for inclusion, data extraction was performed, which included information such as authors, publication date, sample size, age, treatment duration, interventions, and outcome measures. The extracted data were entered into an Excel spreadsheet. Any disagreements were discussed with a third researcher for resolution.

### 2.5. Quality assessment of the studies

Two researchers independently assessed the quality of the included studies using the Cochrane risk of bias assessment tool from the Cochrane Intervention Review Handbook (version 5.0.2). The evaluation included random sequence generation, allocation concealment, blinding implementation, completeness of data, selective reporting of outcomes, and other potential biases. Each item was rated as “unclear,” “low risk,” or “high risk.”

### 2.6. Statistical methods

Stata 14.0 software was used to analyze the outcome measures of the included studies. Categorical data or binary variables were expressed as odds ratios (OR) with 95% confidence intervals (CI), while continuous data or standardized mean differences were used for continuous variables, with 95% CI. Heterogeneity was assessed using the *Q*-test and *I*^2^ statistic. If *P* >.1 or *I*^2^ <50%, homogeneity was assumed, and a fixed-effect model was used. If *P* <.1 or *I*^2^ >50%, significant heterogeneity was indicated, and sensitivity analysis was performed by excluding individual studies or subgroup analysis to explore the sources of heterogeneity. If the source of heterogeneity could not be determined, a random-effects model was used.

## 3. Results

### 3.1. Literature screening process

**Figure 1.**
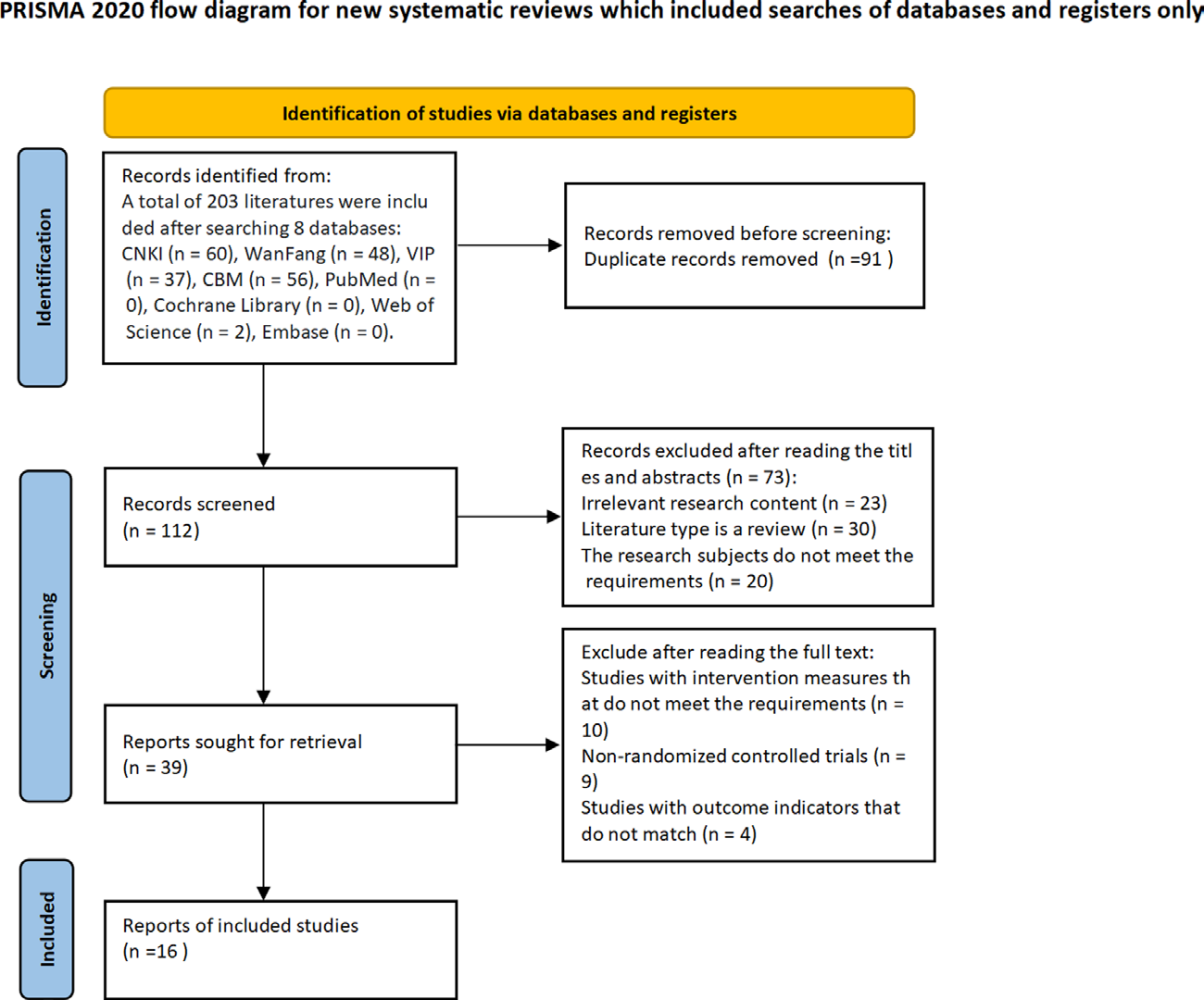
Literature screening flowchart.

A total of 203 articles were initially retrieved. After removing 91 duplicate articles and screening titles and abstracts, 73 studies that did not meet the inclusion criteria were excluded. 39 articles were preliminarily included, and after full-text review, 10 studies with inappropriate interventions (e.g., the experimental group used other TCM formulas instead of Zhizi Qinggan Decoction, or the control group received treatments other than methimazole, such as other Western medications or radioactive iodine, which did not meet the predefined intervention criteria of this study), 9 non-randomized controlled trials, and 4 studies with incompatible outcome indicators (i.e., the included studies either failed to report key predefined outcome measures – such as FT3, FT4, and TSH – or used unvalidated tools to assess outcomes, rendering the data unsuitable for meta-analysis) were excluded. Ultimately, 16 studies were included (Fig. 1).

### 3.2. Basic characteristics of included studies

All included studies were in Chinese, with a total of 16 studies,^[22–37]^ including 1320 hyperthyroidism patients. (Table 1).

**Table 1:** Basic characteristics of the included literature.

Author (year)	Sample size (male/female)		Age		Intervention study		Course of treatment	Outcome indicators
	T	C	T	C	T	C		
Zhang 2017^[22]^	12/18	10/20	28.92 ± 5.61	27. 32 ± 6. 11	A	B	8 wk	FT3, FT4, TSH, clinical effective rate
Yue 2016^[23]^	12/18	11/19	40.8	40.3	A	B	2 mo	Clinical effective rate, adverse effects
Zhang 2018^[24]^	3/17	2/18	37.80 ± 6.13	36.25 ± 7.22	A	B	3 mo	FT3, FT4, TSH, clinical effective rate
Liu 2016^[25]^	60	60	33.6 ± 5.9	33.6 ± 5.9	A	B	3 mo	FT3, FT4, TSH, clinical effective rate, adverse effects
Chen 2016^[26]^	12/37	19/30	36.80 ± 6.95	37.25 ± 6.30	A	B	3 mo	FT3, FT4, TSH, clinical effective rate
Feng 2018^[27]^	19/20	18/21	34.12 ± 2.02	35.12 ± 3.11	A	B	2 mo	Clinical effective rate
Liu 2019^[28]^	15/25	14/26	38. 8 ± 11.7	38. 7 ± 11. 3	A	B	3 mo	FT3, FT4, clinical effective rate, adverse effects, TCM score
Han 2021^[29]^	9/16	10/15	38.28 ± 10.24	37.62 ± 10.13	A	B	3 mo	FT3, FT4, clinical effective rate, adverse effects, TCM score
Xie 2023^[30]^	14/26	16/24	28.99 ± 4.97	28.89 ± 4.93	A	B	8 wk	FT3, FT4, TSH, clinical effective rate, adverse effects, thyroid volume
Cui 2021^[31]^	19/17	21/15	38.61 ± 5.77	38.65 ± 5.82	A	B	3 mo	FT3, FT4, TCM score
Wang 2023^[32]^	38/22	37/23	43.26 ± 5.74	42.75 ± 6.02	A	B	2 mo	FT3, FT4, TSH, clinical effective rate, adverse effects, TCM score
Shi 2024^[33]^	22/26	25/23	47.23 ± 2.18	47.33 ± 2.31	A	B	3 mo	Clinical effective rate, adverse effects, thyroid volume
Wang 2014^[34]^	21/33	22/32	17~65	18~67	A	B	6 mo	Clinical effective rate, adverse effects
Zhang 2021^[35]^	28/31	30/29	48.06 ± 2.79	48.08 ± 2.84	A	B	2 mo	FT3, FT4, TSH, clinical effective rate, thyroid volume
Li 2021^[36]^	40	40	30~50	31~50	A	B	3 mo	Clinical effective rate, adverse effects
Wang 2021^[37]^	14/16	13/17	52.60 ± 4.63	49.30 ± 3.21	A	B	3 mo	FT3, FT4, TSH, clinical effective rate,

A = control group + Zhizi Qinggan decoction, B = methimidazole tablets, C = control group, FT3 = free triiodothyronine, FT4 = free thyroxine, T = test group, TSH = thyroid-stimulating hormone.

### 3.3. Quality assessment of included studies

**Figure 2.**
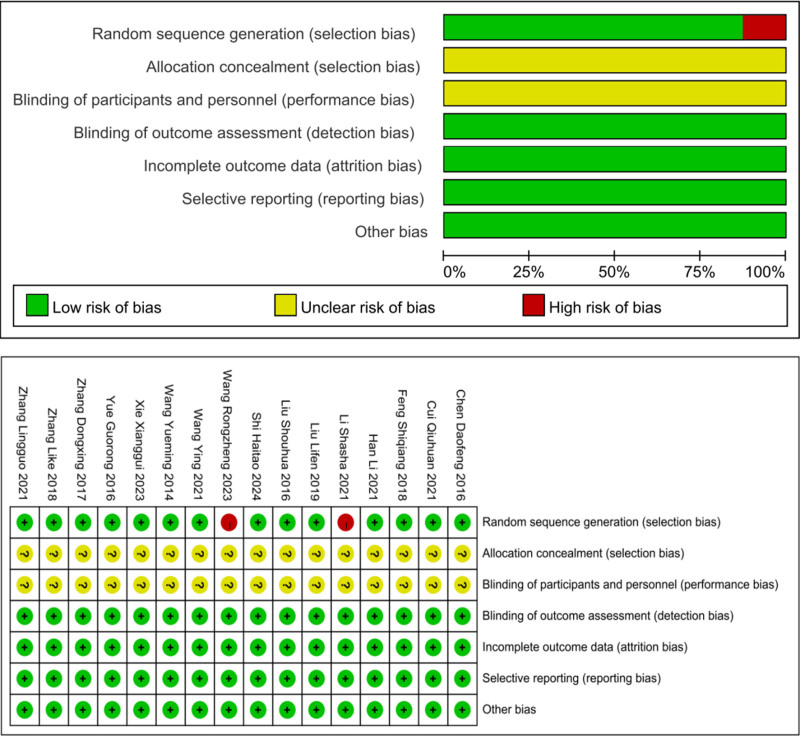
Bias risk assessment of included studies.

Six studies^[22,25,30,31,33,37]^ used random number tables for randomization, 1 study^[27]^ used computer-based random grouping, and 2 studies^[32,36]^ did not mention the allocation concealment method and were rated as “high risk.” The remaining 7 studies reported using random methods for group allocation. Additionally, all 16 studies mentioned the implementation of allocation concealment and blinding of patients, interventionists, and outcome assessors, leading to an “unclear” risk of bias rating. None of the 16 studies had patient dropouts, so they were rated as “low risk.” All 16 studies reported results for all observed outcomes, thus rated as “low risk” as well (Fig. 2).

### 3.4. Meta-analysis results

#### 3.4.1. Primary outcome measures

##### 3.4.1.1. Free triiodothyronine levels

**Figure 3.**
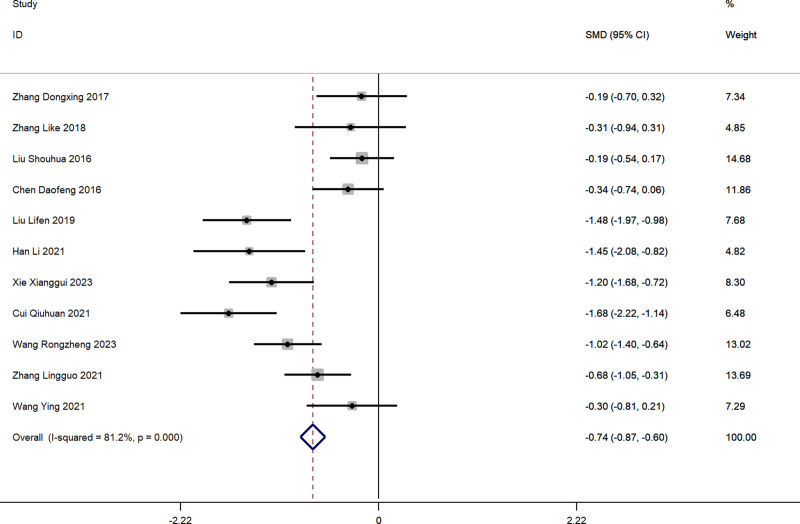
Forest plot comparing FT3 levels between control and experimental groups. FT3 = free triiodothyronine.

Eleven studies^[22,24–26,28–32,35,37]^ reported on FT3 levels, including a total of 898 patients. Comparing FT3 levels after treatment, significant heterogeneity was found among the studies. Even after removing individual studies, heterogeneity remained high (*P* <.001, *I*² = 81.2%). A random-effects model was used for the meta-analysis, which showed that the experimental group improved FT3 levels in hyperthyroidism patients significantly better than the control group (SMD = −0.79, 95% CI [−1.11 to −0.47], *P* <.01) (Fig. 3).

##### 3.4.1.2. Free thyroxine levels

**Figure 4.**
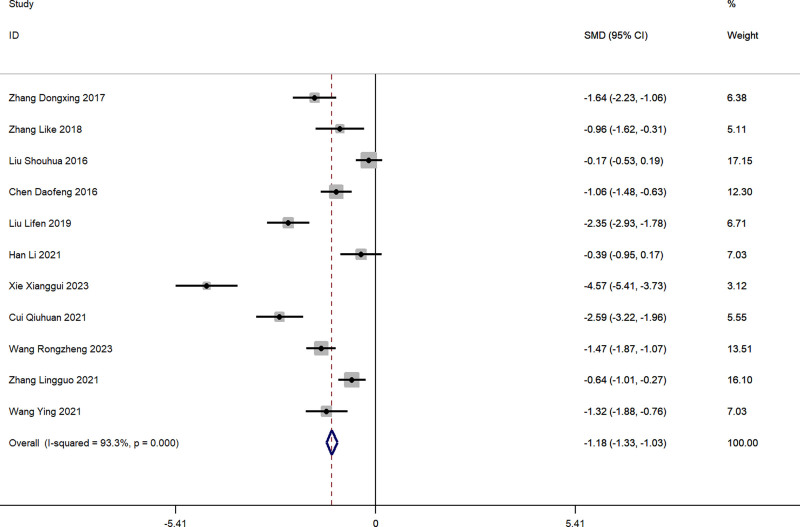
Forest plot comparing FT4 levels between control and experimental groups. FT4 = free thyroxine.

Eleven studies^[22,24–26,28–32,35,37]^ reported on FT4 levels, including a total of 898 patients. Comparing FT4 levels after treatment, heterogeneity was found among the studies. Even after removing individual studies, heterogeneity remained high (*P* <.001, *I*² = 96.1%). A random-effects model was used for the meta-analysis, which showed that the experimental group improved FT4 levels in hyperthyroidism patients significantly better than the control group [SMD = −2.09, 95% CI (−2.91 to −1.28), *P* <.01] (Fig. 4).

##### 3.4.1.3. Thyroid-stimulating hormone levels

**Figure 5.**
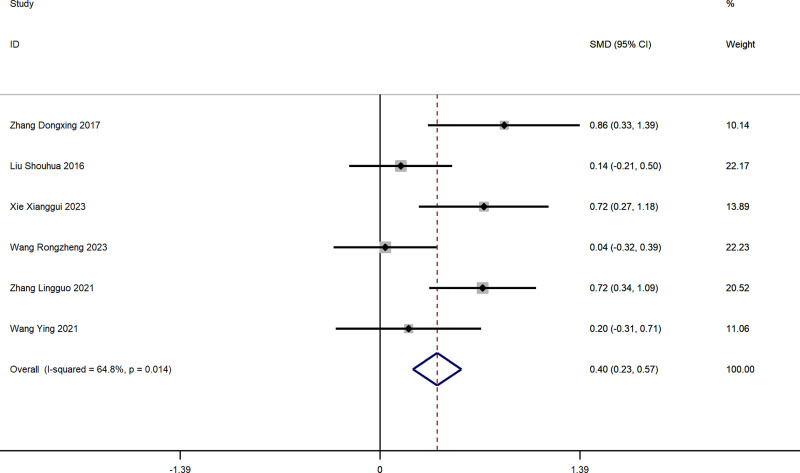
Forest plot comparing TSH levels between control and experimental groups. TSH = thyroid-stimulating hormone.

Six studies^[22,25,30,32,35,37]^ reported on TSH levels, including a total of 438 patients. Comparing TSH levels after treatment, heterogeneity was found among the studies. Even after removing individual studies, heterogeneity remained (*P* = .014, I² = 64.8%). A random-effects model was used for the meta-analysis, which showed that the experimental group significantly reduced TSH levels in hyperthyroidism patients compared to the control group [SMD = 0.40, 95% CI (0.23–0.57), *P* <.01] (Fig. 5).

##### 3.4.1.4. Clinical total efficacy rate

**Figure 6.**
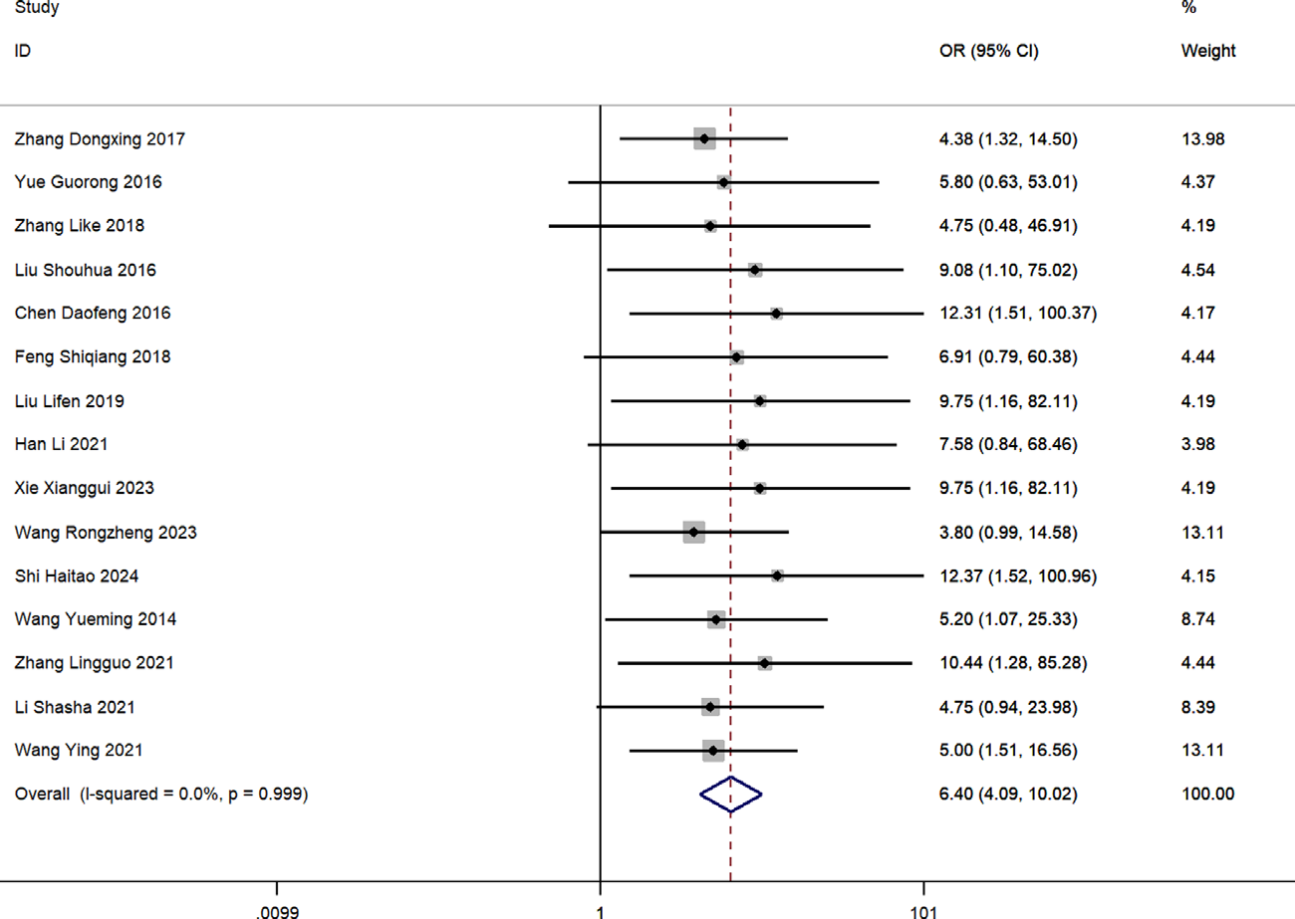
Forest Plot comparing clinical total effective rate between control and experimental groups.

Fifteen studies^[22–30,32–37]^ reported on clinical efficacy, including a total of 1248 patients. The results showed homogeneity across studies (*P* = .999, I² = 0%), and a fixed-effect model was used, which indicated that the experimental group achieved a significantly higher clinical efficacy in treating hyperthyroidism patients than the control group [OR = 6.05, 95% CI (3.85–9.51), *P* <.01] (Fig. 6).

#### 3.4.2. Secondary outcome measures

##### 3.4.2.1. Adverse reactions

**Figure 7.**
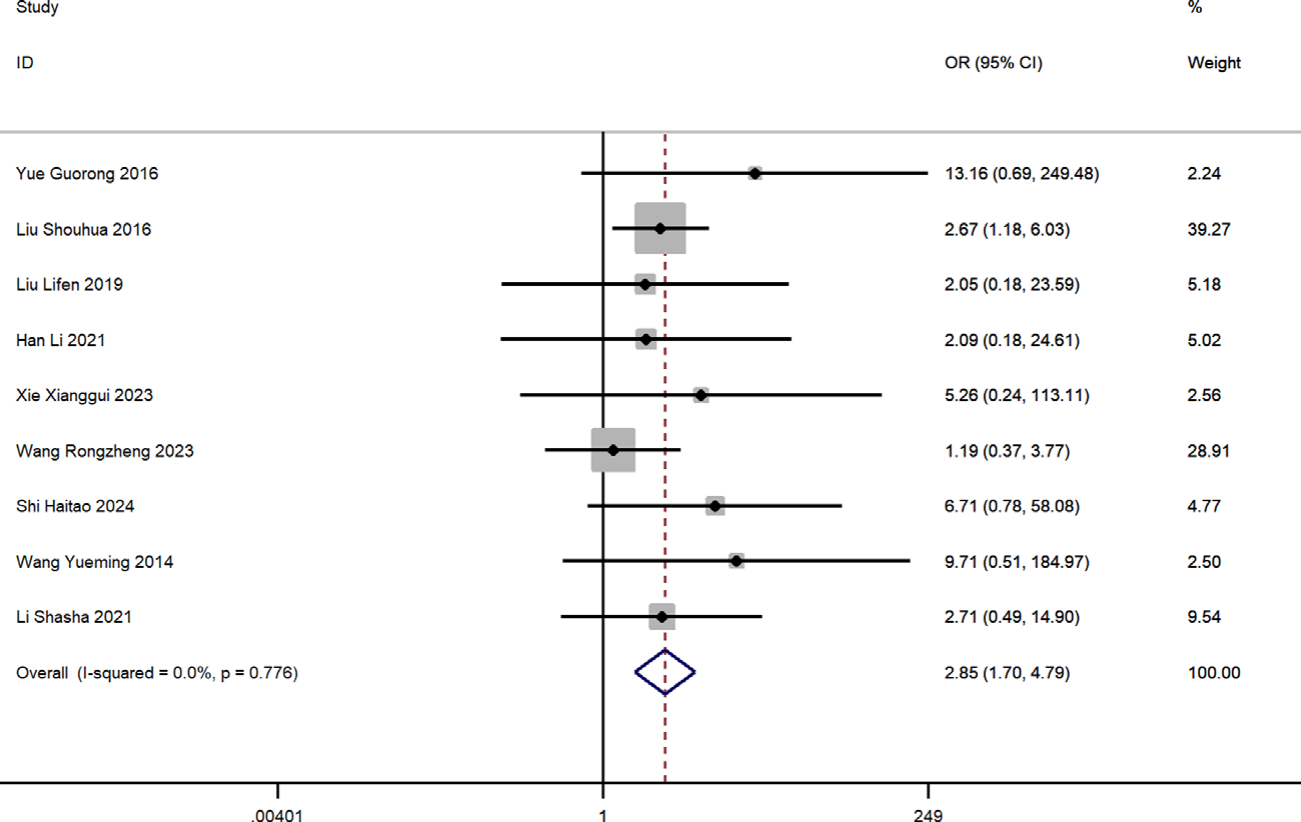
Forest plot comparing adverse reactions between control and experimental groups.

Nine studies^[23,25,28–30,32–34,36]^ reported on adverse reactions, including a total of 794 patients. The results showed homogeneity among studies (*P* = .787, I² = 0%), and a fixed-effect model indicated that the experimental group significantly reduced adverse reactions in hyperthyroidism patients compared to the control group [OR = 2.61, 95% CI (1.52–4.45), *P* <.001] (Fig. 7).

##### 3.4.2.2. Thyroid volume

**Figure 8.**
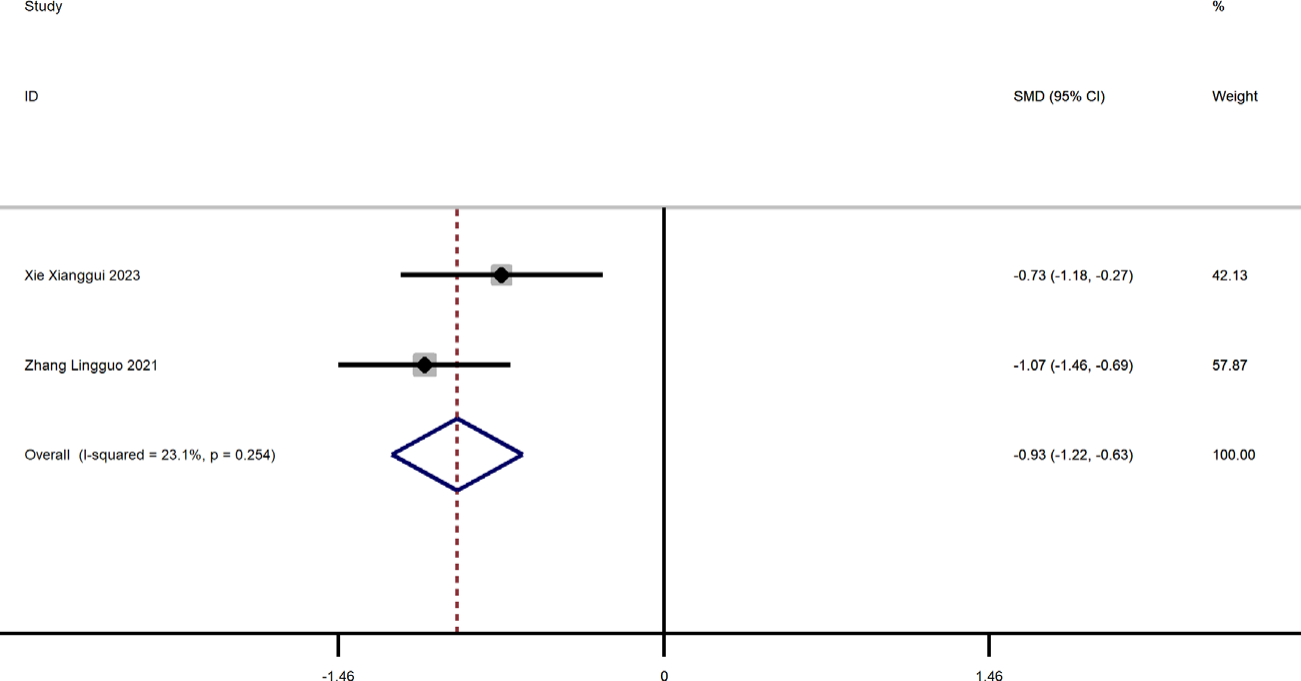
Forest plot comparing thyroid volume between control and experimental groups.

Three studies^[30,33,35]^ reported on thyroid volume, including a total of 294 patients. Significant heterogeneity was found among studies (*P* = .02, I² = 83.3%). After removing individual studies, it was found that the study by Shi Haitao^[33]^ was the source of heterogeneity. After excluding this study (*P* = .254, I² = 23.1%), a fixed-effect model was used for analysis, which showed that the experimental group significantly improved thyroid volume in hyperthyroidism patients compared to the control group [SMD = −0.92, 95% CI (−1.26 to −0.58), *P* <.01] (Fig. 8).

##### 3.4.2.3. Traditional Chinese medicine syndrome score

**Figure 9.**
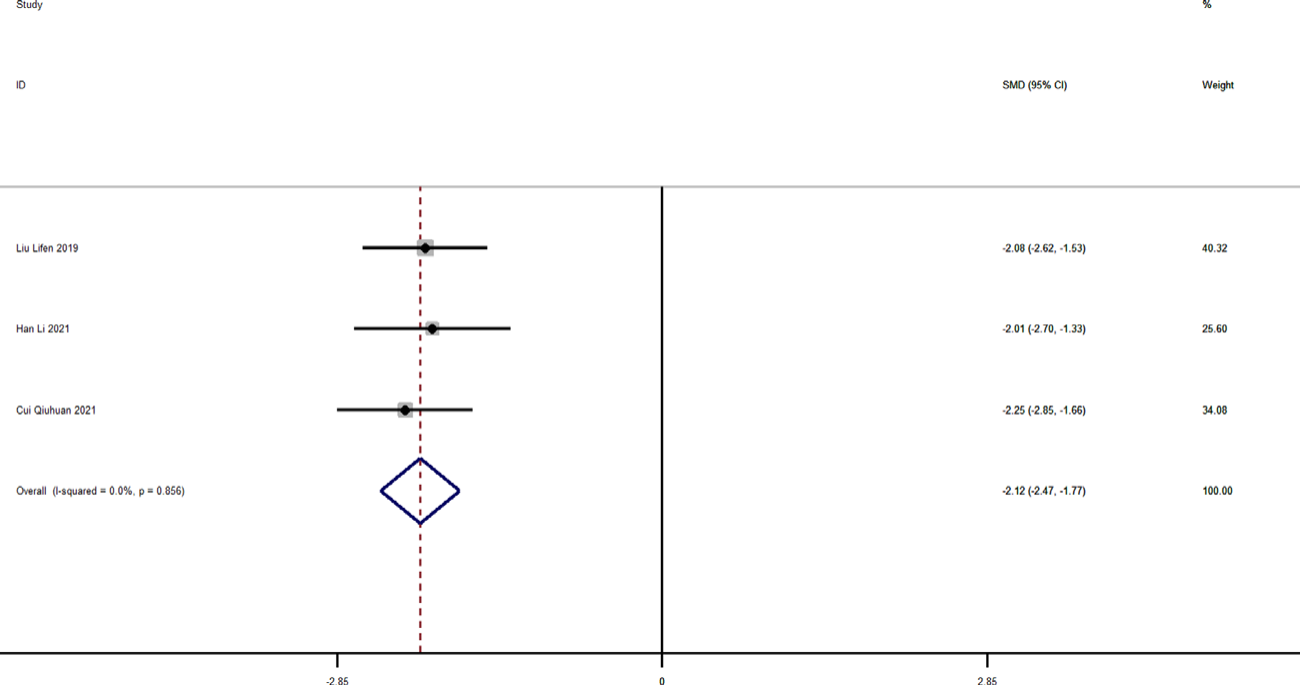
Forest plot comparing traditional Chinese medicine syndrome scores between control and experimental groups.

Four studies^[28,29,31,32]^ reported on TCM syndrome scores, including a total of 322 hyperthyroidism patients. Comparing TCM syndrome scores after treatment, significant heterogeneity was found (*P* = .004, I² = 77.3%). After excluding individual studies, it was found that the study by Wang Rongzheng^[25]^ was the source of heterogeneity, due to a 2-month intervention period, whereas the other 3 studies had a 3-month intervention period. After excluding this study (*P* = .856, I² = 0.0%), a fixed-effect model was used for analysis, which showed that the experimental group significantly improved TCM syndrome scores in hyperthyroidism patients compared to the control group [SMD = −2.12, 95% CI (−2.47 to −1.47), *P* <.01] (Fig. 9).

### 3.5. Subgroup analysis

**Figure 10.**
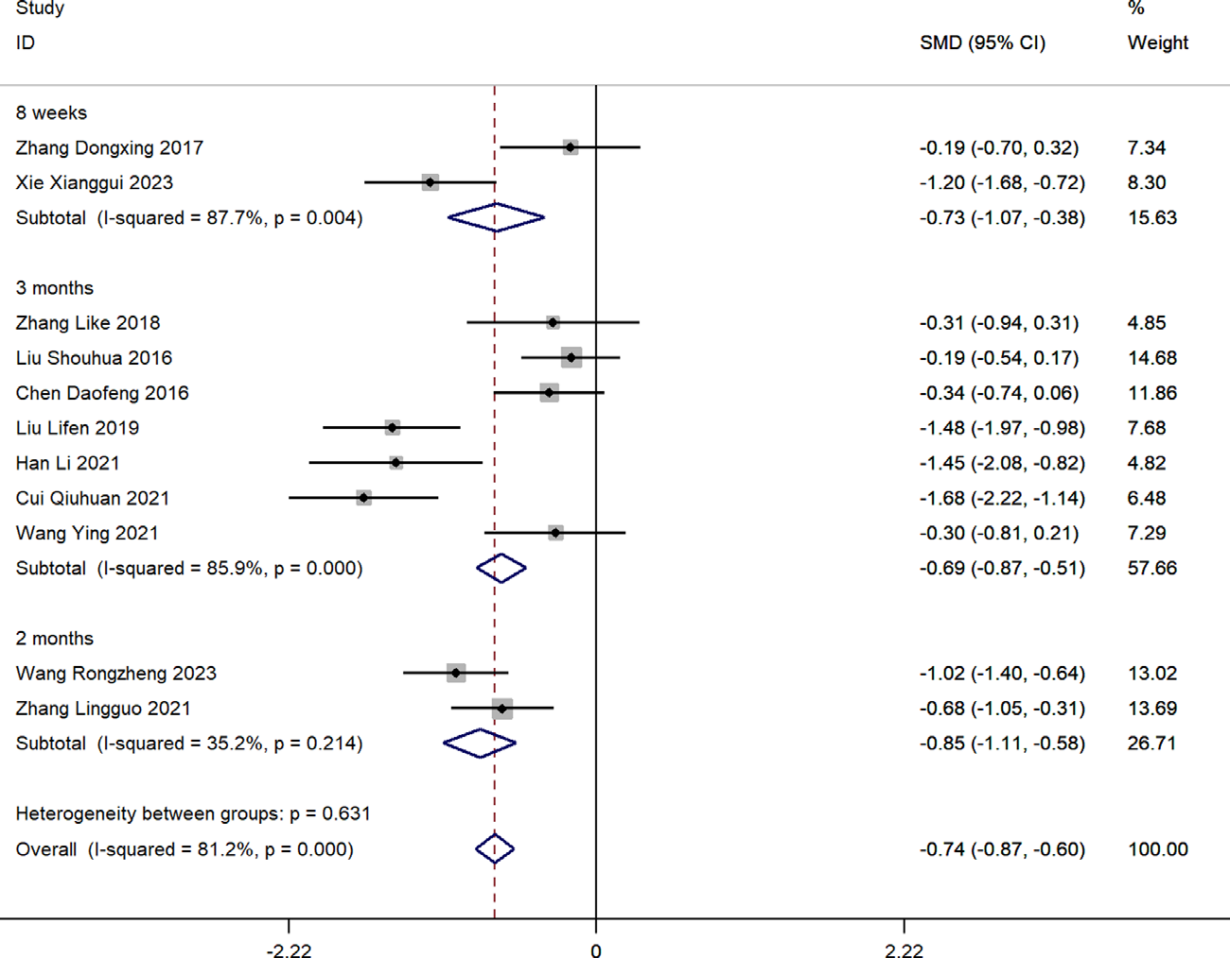
Subgroup analysis forest plot for FT3. FT3 = free triiodothyronine.

**Figure 11.**
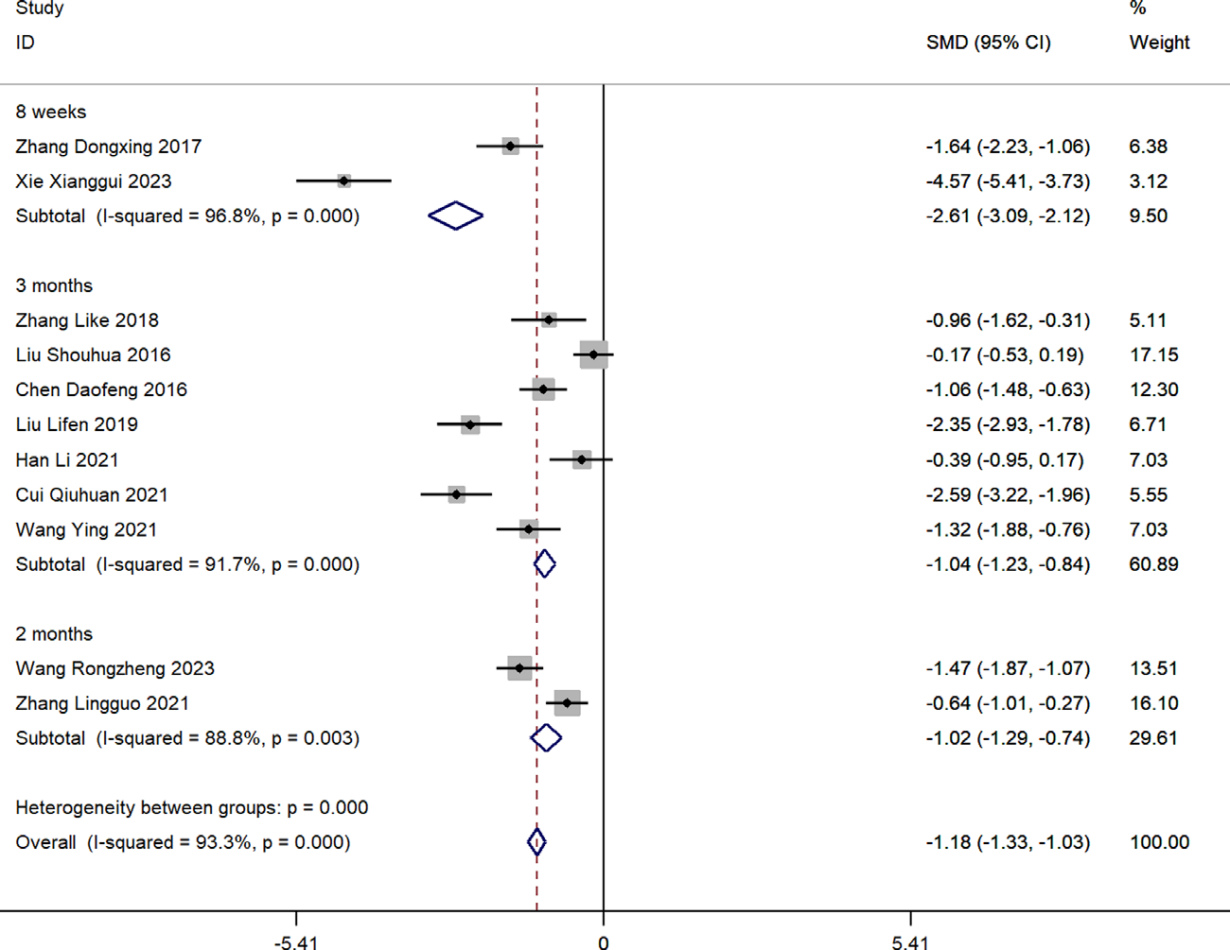
Subgroup analysis forest plot for FT4. FT4 = free thyroxine.

**Figure 12.**
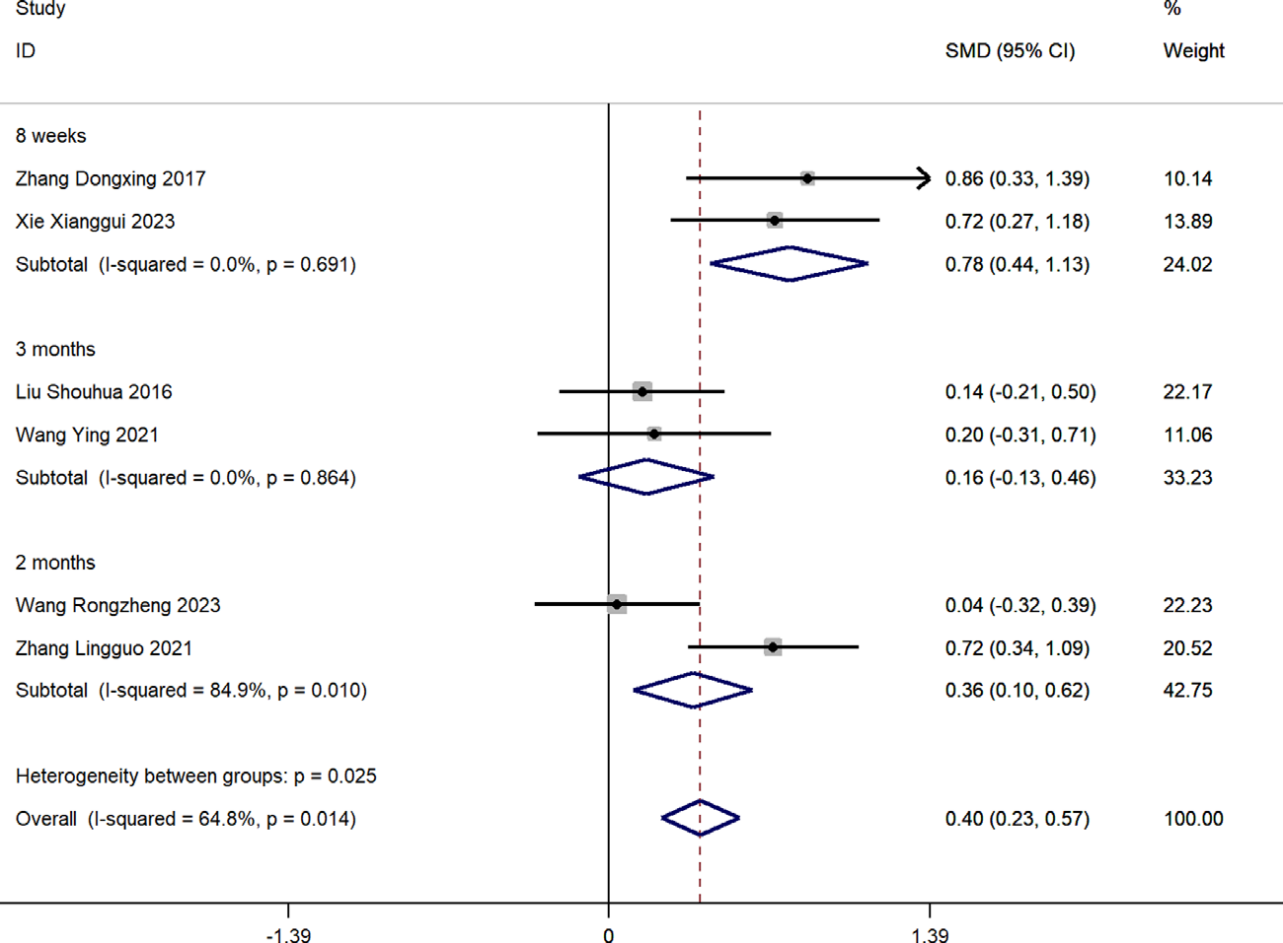
Subgroup analysis forest plot for TSH. TSH = thyroid-stimulating hormone.

To further explore the robustness of the primary outcome measures and potential sources of heterogeneity, subgroup analyses of FT3, FT4, and TSH were performed according to the intervention duration of the experimental group as the grouping condition. The results showed that the heterogeneity of FT3 could be eliminated (I²=35.2%, *P* = .214), but the heterogeneity of FT4 and TSH could not be eliminated (Figs. 10–12).

### 3.6. Publication bias

**Figure 13.**
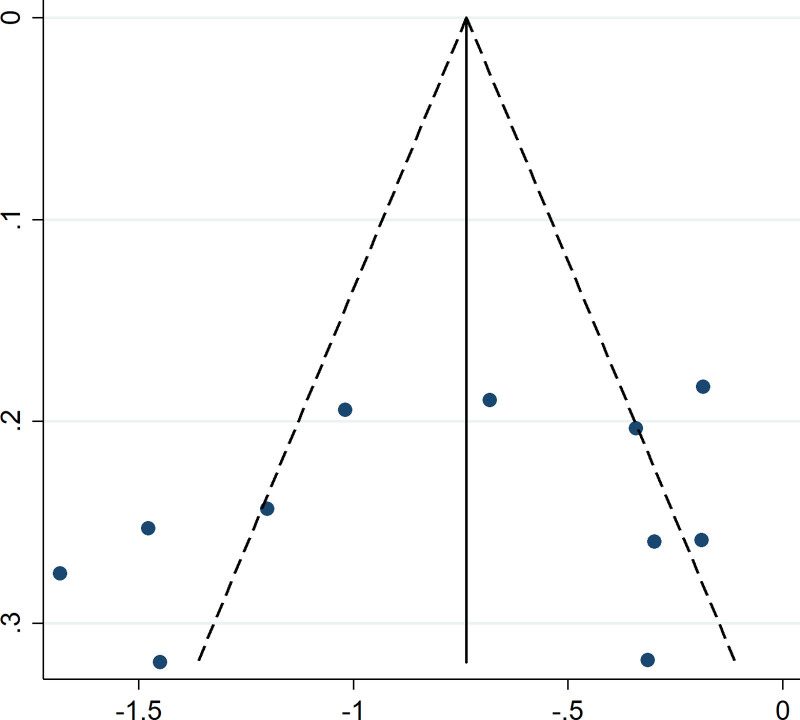
Funnel plot for FT3. FT3 = free triiodothyronine.

**Figure 14.**
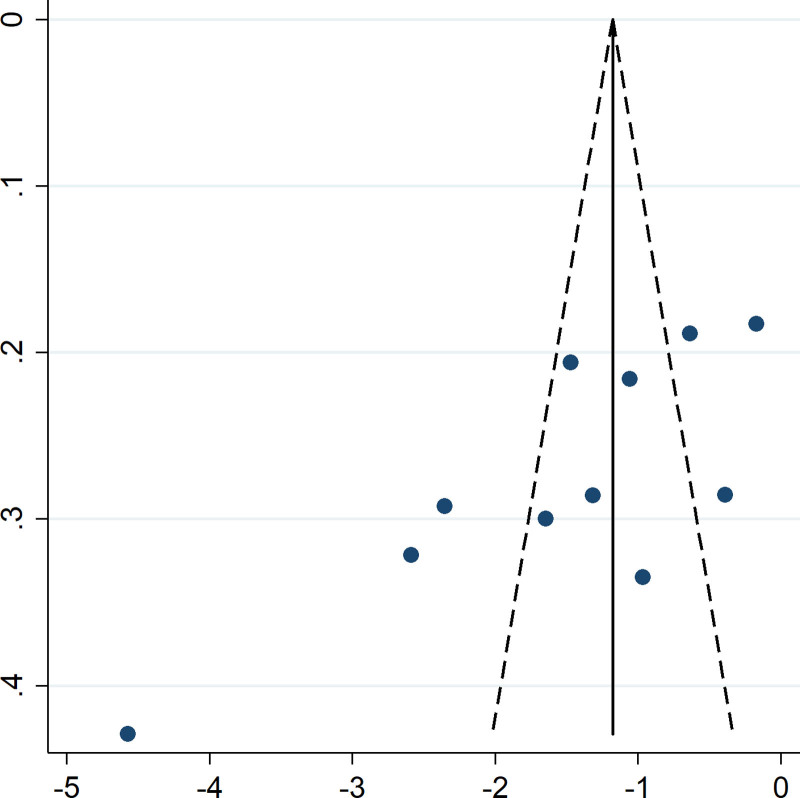
Funnel Plot for FT4. FT4 = free thyroxine.

**Figure 15.**
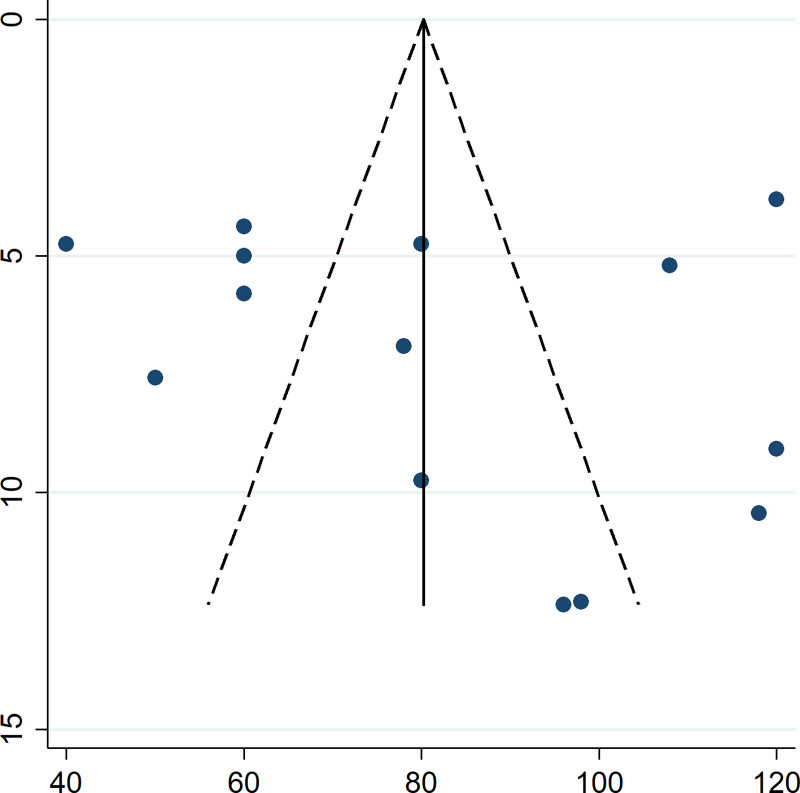
Funnel plot for clinical total effective rate.

According to the Cochrane Intervention Review Handbook, funnel plots for publication bias assessment can only be used when the number of included studies exceeds 10. Publication bias was analyzed for outcome measures (FT3, FT4, total efficacy rate) with more than 10 included studies. The results indicated an asymmetrical distribution, suggesting the possibility of publication bias (Figs. 13–15).

## 4. Discussion

Hyperthyroidism is a common endocrine disorder, and its pathogenesis is still unclear in Western medicine. While treatment methods are diverse, the onset of adverse reactions is frequent, and relapse after treatment is common. In contrast, TCM has a long history in treating hyperthyroidism with fewer side effects and unique advantages. In recent years, increasing studies have shown that the combination of Chinese and Western medicines reduces the incidence of side effects and lowers the risk of relapse in patients with hyperthyroidism.^[38]^ Recent data show that the high-polar iridoid glycosides in gardenia significantly reduce thyroid hormone (T3 and T4) levels in hyperthyroid rats’ serum and restore thyroid function by regulating the expression of genes related to thyroid hormone synthesis, such as deiodinase and thyroid peroxidase.^[39,40]^ In addition, Feng et al.^[41]^ suggested that gardenia may indirectly affect the secretion of TSH by regulating the signaling pathway of the hypothalamic-pituitary-thyroid axis (HPT axis), thereby regulating the synthesis and release of thyroid hormones.

This study followed the Cochrane systematic review guidelines and conducted a comprehensive analysis of 16 RCTs to evaluate the efficacy of Zhizi Qinggan Decoction combined with Methimazole Tablets in the treatment of hyperthyroidism. The meta-analysis results indicate that Zhizi Qinggan Decoction achieves a higher overall efficacy rate in the treatment of hyperthyroidism compared to the control group. For example, our results show reduced FT3, FT4, and TSH levels, attenuated thyroid volume and adverse reactions, and improved TCM syndromes, thus enhancing core outcomes in patients with hyperthyroidism compared to the control group. Further subgroup analysis based on intervention duration was conducted to explore the heterogeneity of the efficacy of Zhizi Qinggan Decoction. The results showed that the efficacy effect of Zhizi Qinggan Decoction remained consistent across all subgroups. This indicates the stable therapeutic effect of Zhizi Qinggan Decoction in treating hyperthyroidism, providing reliable evidence for clinical individualized treatment. At the same time, the consistency of the efficacy direction suggests that different patient characteristics and treatment regimens do not significantly weaken its therapeutic effects. This further confirms the stability and reliability of the clinical efficacy results.

This study also has some limitations: (1) Most of the included studies are clinical controlled trials with relatively limited sample sizes; (2) Some indicators exhibit significant heterogeneity, and although attempts have been made to resolve this through sensitivity analysis, it may still affect the accuracy of the results; (3) The included studies did not report the standardized dosage of Zhizi Qinggan Decoction (for example, differences in the weight of key medicinal materials such as Gardenia jasminoides and Bupleurum chinense, or variations in administration frequency). Therefore, it is impossible to assess the potential dose-response relationship between this decoction and hormone levels or other outcome indicators; (4) Some of the testing methods used for primary outcome indicators are susceptible to individual patient differences and subjective factors, such as self-assessment of symptoms, which may introduce measurement bias; (5) All patients included in this study are Asians, so the research conclusions may be more applicable to Asian and Chinese adult populations; however, the applicability to other ethnic groups or populations remains to be further explored and verified. (6) Hormones such as FT3, FT4, and TSH are significantly affected by circadian variations, participant characteristics, participant age, and hormone detection methods, yet the included literature fails to account for or explain such issues.

## 5. Conclusion

This meta-analysis provides a comprehensive evaluation of the effect of Zhizi Qinggan Decoction in treating hyperthyroidism. The results show that, compared with the control group treated with Methimazole tablets alone, the combination of Zhizi Qinggan Decoction and Methimazole tablets improves clinical symptoms related to thyroid function and regulates thyroid function, with a trend of reducing the incidence of adverse reactions associated with Western medicine. However, current studies have limitations such as small sample sizes and varying quality. Overall, Zhizi Qinggan Decoction shows significant potential in the treatment of hyperthyroidism, and future high-quality, large-sample studies are needed to further verify its exact efficacy and mechanism of action, providing stronger evidence for its clinical application.

## Author contributions

**Conceptualization:** Min Zhang.

**Data curation:** Jiashuai Chu.

**Investigation:** Chang Xiu.

**Software:** Jing Bai.

**Supervision:** Lei Fan.

**Validation:** Jiashuai Chu.

**Visualization:** Jiashuai Chu.

**Writing – original draft:** Jiashuai Chu.

**Writing – review & editing:** Jiashuai Chu.

## Supplementary Material


