# Supplementary material for: The effect of Zhizi Qinggan decoction in treating hyperthyroidism: A systematic review and meta-analysis
Source: Medicine (Baltimore). 2025 Sep 12;104(37):e44483. doi: 10.1097/MD.0000000000044483 (PMC12440526; doi:10.1097/MD.0000000000044483)
Supplement: Supplementary file 1 [file medi-104-e44483-s001.docx]

Supplementary file 1. The English search formula takes PubMed as an example.

#1 Hyperthyroidism[MeSH]

#2 Hyperthyroid[Ti/Ab] OR Hyperthyroids[Ti/Ab] OR Primary Hyperthyroidism[Ti/Ab] OR Graves Disease[Ti/Ab]

#3 #1 OR #2

#4 Zhi Zi Qing Gan Tang[Ti/Ab] OR Zhizi Qinggan Decoction[Ti/Ab] OR zhi zi Decoction[Ti/Ab] OR Gardenia Decoction[Ti/Ab]

#5 #3 AND #4
